# Single Immunoglobulin IL-1-Related Receptor (SIGIRR) Gene rs7396562 Polymorphism and Expression Level in Rheumatoid Arthritis

**DOI:** 10.1155/2021/6683148

**Published:** 2021-05-07

**Authors:** Xiaoke Yang, Mingyue Zhang, Shengqian Xu, Haifeng Pan, Ruixue Leng, Zongwen Shuai

**Affiliations:** ^1^Department of Rheumatology and Immunology, The First Affiliated Hospital of Anhui Medical University, Hefei, Anhui, China; ^2^Inflammation and Immune Mediated Diseases Laboratory of Anhui Province, Hefei, Anhui, China; ^3^Department of Medical Record Room, Medical Affairs, Fuyang Hospital of Anhui Medical University, Fuyang, Anhui, China; ^4^Department of Epidemiology and Biostatistics, School of Public Health, Anhui Medical University, Hefei, Anhui, China

## Abstract

**Objectives:**

The aim of our study was to investigate the association of single-nucleotide polymorphism (SNP) and mRNA expression profile of *single immunoglobulin IL-1-related receptor* (*SIGIRR*) in rheumatoid arthritis (RA) in a Chinese population.

**Methods:**

*SIGIRR rs7396562* polymorphism was genotyped using TaqMan allelic discrimination assay in 517 RA patients and 601 healthy controls. Simultaneously, the SIGIRR mRNA expression levels of 79 RA patients and 76 healthy controls were examined by real-time quantitative polymerase chain reaction (RT-qPCR).

**Results:**

The frequency of *SIGIRR rs7396562* T allele was significantly higher in RA patients compared with healthy controls (T versus G: OR = 1.277, 95%CI = 1.079 − 1.511, *P* = 0.004). The TT genotype of *SIGIRR* rs7396562 was more frequent in RA patients than in healthy controls (OR = 1.547, 95%CI = 1.107 − 2.163, *P* = 0.011). Moreover, we also found a significant difference in the recessive model (TT versus TG+GG: OR = 1.439, 95%CI = 1.122 − 1.847, *P* = 0.004). However, no significant evidence was observed for the association of the *SIGIRR rs7396562* with RA in dominant model (TT+TG versus GG: OR = 1.275, 95%CI = 0.947 − 1.717, *P* = 0.109). Further analysis showed no association between *SIGIRR rs7396562* polymorphism and laboratory parameters of RA patients (all *P* > 0.05). The mRNA expression of SIGIRR was decreased in PBMCs of patients with RA when compared to healthy controls (*Z* = −2.459, *P* = 0.014). No significant differences in SIGIRR mRNA expression levels were observed in patients with RA with different genotypes (*P* = 0.280).

**Conclusions:**

Our findings demonstrated that the dysregulation of SIGIRR might be associated with the pathogenesis of RA, and *SIGIRR rs7396562* polymorphism might contribute to RA susceptibility in the Chinese population.

## 1. Introduction

Rheumatoid arthritis (RA) is a common chronic inflammatory autoimmune disease which mainly causes joint erosion and destruction and progressive disability [1]. RA affects 0.5-1% of adults worldwide and seems to affect females three times more likely than males [2]. The etiology of RA is still unclear. Although both genetic and environmental factors contribute to its development, the genetic factor may account the principal risk for RA susceptibility [3, 4].

The interleukin-1 receptor (IL-1R) superfamily includes two subfamilies of IL-1R-like receptors (ILRs) and Toll-like receptors (TLRs); both of which are involved in inflammatory response and innate immunity [5]. Single Ig IL-1-related receptor (SIGIRR), also named Toll/IL-1 receptor 8 (TIR8) or IL-1R8, is a new member of the IL-1R superfamily [6]. SIGIRR is widely expressed in many epithelial tissues, with high levels in the liver, kidney, lung, parathyroid, lymphoid organs, digestive tract, and pregnant uterus. As for leukocytes, SIGIRR is expressed by T and B cells, monocytes, NK cells, and dendritic cells (DCs) [7]. SIGIRR contains a TIR intracellular domain and a single extracellular Ig domain. On the one hand, SIGIRR dimerized through the TIR intracellular domain, inducing the recruitment of myeloid differentiation factor 88 (MyD88), which interacted with downstream signaling molecules including TNF receptor-associated factor 6 (TRAF6) and IL-1R-associated kinase 1 (IRAK). On the other hand, SIGIRR extracellular domain was shown to inhibit the dimerization between IL-1R1 and IL-1R3, and participate in the suppression of IL-1R4/ST2 signal transduction [8]. Al-Kuhlani et al. [9] highlighted that SIGIRR was involved in signal transduction via TLRs during chlamydial infection in epithelial cells and downregulated this signaling pathway. Moreover, SIGIRR overexpression inhibited the production of cytokines induced by TLR in monocyte-derived macrophages and DCs, while SIGIRR knockdown led to an increase in cytokine production following TLR stimulation [10]. Furthermore, SIGIRR has been implicated in the inhibition of Th1, Th2, and Th17 immune responses [11–13].

The human *SIGIRR* gene located on chromosome 11p15.5 is organized in 10 exons spanning about 11,700 bp [14]. Single-nucleotide polymorphism (SNP) is a third generation biomarker which plays an important role in exploring the pathogenic mechanisms of diseases. Our previous study has demonstrated that *SIGIRR* gene *rs7396562* was associated with the risk of systemic lupus erythematosus (SLE) in a Chinese population [15]. However, as far as we know, no study has explored the relationship between *SIGIRR* polymorphisms and RA patients. Meanwhile, studies on the SIGIRR expression level in RA are very limited and the results were controversial [10, 16–18]. Thus, in this study, we performed a case-control study to access whether an association existed between *SIGIRR rs7396562* and the susceptibility to RA in a Chinese population. Besides, we also evaluated the SIGIRR mRNA expression levels in peripheral blood mononuclear cells (PBMCs) from RA patients and healthy subjects.

## 2. Materials and Methods

### 2.1. Participants

A total of 517 RA patients (83 males and 434 females, mean age 50.75 ± 13.83 years) and 601 age- and gender-matched healthy controls (112 males and 489 females, mean age 49.13 ± 15.66 years) were consecutively recruited to evaluate the association between *SIGIRR* gene *rs7396562* polymorphism and RA. All these RA patients who fulfilled the 1987 ACR revised criteria were recruited from the First Affiliated Hospital of Anhui Medical University from January 2014 to June 2018 [19]. The exclusion criteria of patients were as follows: (1) patients suspected of alcohol or drug abuse; (2) patients with other autoimmune diseases, cancers, chronic infectious diseases, and serious liver or kidney disease; and (3) patients with severe acute infections within one month prior to admission. All the healthy controls included in our study were free of RA or other autoimmune diseases.

The mRNA expression levels of SIGIRR were detected in 79 RA patients (7 males and 72 females, mean age 52.72 ± 11.48 years) and 76 age- and gender-matched healthy controls (10 males and 66 females, mean age 49.82 ± 11.84 years) randomly selected from the genotyping samples.

The demographic and clinical data were obtained from the hospital records or questionnaire. Informed consent was collected from all participants according to the 1964 Helsinki Declaration. The study protocol has acquired the permission from the Ethics Committee of Anhui Medical University.

### 2.2. SNP Selection

We conducted a systemic search on the previously reported SNPs in *SIGIRR* gene. The eligible SNP selection criteria were as follows: (a) a tagging SNP; (b) the minor allele frequency (MAF) > 0.05 in CHB; and (c) it has been shown to be associated with RA or other autoimmune diseases. Eventually, one tag-SNP located in the intron region of SIGIRR (rs7396562) was selected.

### 2.3. DNA Extraction and Genotyping

5 mL peripheral blood was collected in vacutainer tubes containing EDTA from RA patients and healthy controls. Genomic DNA samples were extracted from peripheral blood leukocytes by the Flexi Gene DNA Kit (Qiagen, Valencia, CA). The genotyping was performed by TaqMan allelic discrimination assay on the EP1 platform (Fluidigm, South San Francisco, CA, USA). The probe assay ID of SIGIRR rs7396562 was C_189316652_10.

### 2.4. Real-Time Quantitative PCR

PBMCs were freshly isolated from peripheral blood by Ficoll-Hypaque density gradient centrifugation. Total RNA was extracted from PBMCs by using TRIzol reagent and reverse-transcribed into cDNA by the PrimeScript RT reagent kit (Takara Bio Inc., Japan).

RT-qPCR with SYBR Green (SYBR® Premix Ex Taq™ II, Takara Bio Inc., Japan) was conducted on the ABI ViiA™ 7 Real-Time PCR System (Applied Biosystems, Foster City, CA, USA) to compare the mRNA expression of SIGIRR gene (forward primer: 5′-CTCCCCGTCTGAAGACCAG-3′, reverse primer: 5′-CCCCAATTCCCAATGGAAGC-3′) and housekeeping gene *β*-actin (forward primer: CACGAAACTACCTTCAACTCC, reverse primer: CATACTCCTGCTTGCTGATC). The primers were designed by the National Center for Biotechnology Information (NCBI) and Primer Bank (https://pga.mgh.harvard.edu/primerbank/). The RT-qPCR reactions contained 0.4 *μ*L forward primer and 0.4 *μ*L reverse primer (10 *μ*M). Cycle conditions were as follows: 95°C for 1 min, followed by 42 cycles at 95°C for 10 sec, 60°C for 30 sec, and 72°C for 1 min. The relative expression levels were computed using the 2^−ΔΔCt^.

The quality control measurements applied for the molecular experiment were as follows: (1) The experiment process is carried out strictly according to the laboratory operation rules; (2) All the reagents are operated according to the instruction in the reagent kit, and the experiment operation should be standardized; (3) Before the formal experiment, a preliminary experiment is carried out to find out the best experimental conditions. After the experimental conditions are stable, a formal experiment is carried out; (4) Each experiment was carried out by the same operator under the same conditions as possible; and (5) The results of the experiment are read by two professional technicians.

### 2.5. Statistical Analysis

The differences in categorical variables between different groups were compared by the chi-square (*χ*^2^) test. Odds ratio (OR) and 95% confidence interval (95% CI) were also calculated. In addition, the *χ*^2^ test was also used to determine whether the frequency of genotypes was consistent with Hardy-Weinberg equilibrium (HWE). The Power and Sample Size Calculation Software (version 3.1.2) was applied to calculate the statistical power.

For gene expression study, DAS28 was used to determine the RA activity and stratified according to disease activity (DAS28 ≥ 2.6) or disease in remission (DAS28 ≤ 2.6). The nonparametric Mann-Whitney *U* test was used to compare gene expression between different groups. The correlation between different groups was analyzed by the Spearman rank correlation coefficient test.

All statistical analyses were performed by SPSS 19.0 (IBM Corp). A *P* value less than 0.05 was regarded as statistically significant.

## 3. Results

### 3.1. Basic Characters

The demographic characteristics and laboratory parameters of RA patients are summarized in Table 1. The listed laboratory parameters were anticyclic citrullinated peptide (anti-CCP) (80.7%) and rheumatoid factor (RF) (76.9%).

### 3.2. *SIGIRR* Polymorphisms and Risk of RA

The frequency of genotypes of *SIGIRR rs7396562* was in accordance with HWE in controls (*χ*^2^ = 0.285, *P* = 0.593). The power of the current study was 92.1% for *SIGIRR* rs7396562 to detect a 1.5-fold increased risk at the significance level of 0.05.

As shown in Table 2, the TT genotype of *SIGIRR rs7396562* was more frequent in RA patients than in healthy controls (OR = 1.547, 95%CI = 1.107 − 2.163, *P* = 0.011). Allele contrast showed that the frequency of *SIGIRR rs7396562* T allele was significantly higher in RA patients compared with healthy controls (T versus G: OR = 1.277, 95%CI = 1.079 − 1.511, *P* = 0.004). Moreover, we also detected a significant difference in the recessive model (TT versus TG+GG: OR = 1.439, 95%CI = 1.122 − 1.847, *P* = 0.004). However, no significant evidence was observed in the dominant model (TT+TG versus GG: OR = 1.275, 95%CI = 0.947 − 1.717, *P* = 0.109).

We also conducted stratification analysis between positive and negative patients in anti-CCP and RF. However, no association of *SIGIR*R *rs7396562* polymorphism with anti-CCP and RF in RA patients was found (Table 3).

### 3.3. SIGIRR mRNA Expression Levels and RA

**Figure 1:**
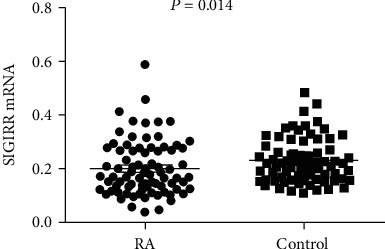
The comparison of SIGIRR mRNA expression levels between RA patients and healthy control.

Gene expression analyses indicated that SIGIRR mRNA expression levels were significantly decreased in PBMCs of RA patients when compared to normal controls (*Z* = −2.459, *P* = 0.014) (Figure 1). However, there were no significant differences in SIGIRR mRNA expression levels between anti-CCP-positive patients and anti-CCP-negative patients, as well as RF-positive patients and RF-negative patients (all *P* > 0.05). Further analysis showed that there were also no significant correlations of SIGIRR mRNA expression levels and CRP, ESR, and DAS28 (all *P* > 0.05).

### 3.4. Association of SIGIRR mRNA Expression Levels with Genotypes in Patients with RA

**Figure 2:**
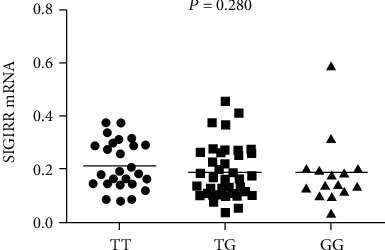
SIGIRR mRNA expression levels in different genotypes of rs7396562 in patients with RA.

In this study, we also examined whether the possible associated genotypes of rs7396562 might be associated with SIGIRR mRNA expression levels. However, no significant differences in SIGIRR mRNA expression levels were observed in RA patients with different genotypes (*P* = 0.280) (Figure 2).

## 4. Discussion

Increasing evidence proved that SIGIRR could be an essential regulator of inflammation and innate and adaptive immune responses [5–15]. Recent studies have demonstrated that SIGIRR levels are upregulated in PBMCs and synovial cells of RA patients and involved in the pathogenesis of this disease [10, 16, 17]. Similar studies have also been conducted among other inflammatory and autoimmune diseases such as SLE and psoriatic arthritis (PsA). A previous report suggested that the frequency of SIGIRR-positive CD4^+^ T cells was reduced in peripheral blood of SLE patients when compared with healthy controls [20]. In addition, Zhu et al. [21] discovered an increased expression of SIGIRR in B cells of SLE patients. PsA patients had lower level of SIGIRR in peripheral blood than healthy donors. Moreover, the lower SIGIRR expression of PsA patients could influence the severity of this disease [22]. Therefore, SIGIRR gene polymorphisms might affect its expression and activity, and thereby involved in RA pathogenesis.

The current study was undertaken to analyze the presence of the *SIGIRR rs7396562* polymorphism in a Chinese population affected by RA. As far as we know, this is the first study demonstrating an association of SNP *rs7396562* with the risk of RA. Our observations suggested that *SIGIRR rs7396562* was a novel genetic marker significantly associated with RA. Interestingly, our previous study also indicated that the genetic variant of *SIGIRR* (*rs7396562*) was correlated with susceptibility to SLE [15]. RA and SLE are the most common autoimmune diseases. The recent GWAS has proposed some common genetic risk components for autoimmune diseases [23].

Subsequently, the SIGIRR mRNA expression in PBMCs of RA and healthy controls was analyzed. We found that the SIGIRR mRNA expression in RA patients was downregulated as compared to healthy controls, which suggested the possibility that SIGIRR is involved in the pathogenesis of RA. SNP *rs7396562* studied in this study was located in the intron region of *SIGIRR* gene. Recent evidence has illustrated that introns contain important gene regulatory sequences with multiple functional roles, including RNA editing, noncoding RNAs, translation elements, and selectable promoter enhancers [24]. Additionally, intron mutations may exert their phenotypic effects by altering gene expression levels [25]. Indeed, we searched for cis-eQTL in eQTL base and found that *rs7396562* loci are cis-eQTL in peripheral blood (Table 4). Therefore, the potential relationship of *SIGIRR rs7396562* SNP and mRNA expression levels was evaluated. Unfortunately, no significant differences in SIGIRR mRNA expression levels were observed in RA patients with different genotypes. This discrepancy may be due in part to insufficient sample size. As a common susceptible site, the most possible mechanism of SIGIRR was it influenced the signal transduction of IL-1R and TLR, thus leading to the alteration in T cell differentiation and activation.

This is the first time to demonstrate that *SIGIRR* gene *rs7396562* polymorphism might contribute to RA susceptibility in a Chinese population. However, this study has several limitations which should be mentioned here. First, our study is a hospital-based case-control study, and selection bias was inevitable. Second, we are not able to rule out the potential influence of environmental factor. Third, we only testified one genetic variant (*rs7396562*) of *SIGIRR* and it is still a long way from understanding the entire gene with RA susceptibility. Finally, we have not yet conducted a functional study of SIGIRR.

Taken together, our observations suggested that the dysregulation of SIGIRR might be associated with the pathogenesis of RA, and *SIGIRR* gene *rs7396562* polymorphisms might contribute to RA susceptibility in a Chinese population. Further studies with more sample size on the exact mechanism of SIGIRR in RA could be conducted.

## Figures and Tables

**Table 1:** The demographic characteristics and laboratory parameters of RA patients.

Characteristics	RA patients (*N* = 517)
Age (years), mean ± SD	50.75 ± 13.83
Sex, female/male	434/83
Age of onset (years), M ± SD	44.14 ± 13.89
Disease duration (years), M ± SD	7.08 ± 7.49
Treatment duration (years), mean ± SD	5.94 ± 7.27
Anti-CCP-positive no. (%)	417 (80.7)
RF-positive no. (%)	398 (76.9)

RA: rheumatoid arthritis; RF: rheumatoid factor; Anti-CCP: anticyclic citrullinated peptide.

**Table 2:** Allele and genotype frequencies of *SIGIRR rs739656*2 polymorphism in RA patients and controls.

SNPs	Genotype	RA (*n* = 517), *n* (%)	Controls (*n* = 601), *n* (%)	OR (95% CI)	*P* value
rs7396562	TT	196 (37.9)	179 (29.8)	1.547 (1.107-2.163)	0.011^∗^
	TG	229 (44.3)	292 (48.6)	1.108 (0.806-1.523)	0.527
	GG	92 (17.8)	130 (21.6)	Reference	
	T	621 (60.1)	650 (54.1)	1.277 (1.079-1.511)	0.004^∗^
	G	413 (39.9)	552 (45.9)	Reference	
Dominant model	TT+TG	425 (82.2)	471 (78.4)	1.275 (0.947-1.717)	0.109
	GG	92 (17.8)	130 (21.6)	Reference	
Recessive model	TT	196 (37.9)	179 (29.8)	1.439 (1.122-1.847)	0.004^∗^
	TG+GG	321 (62.1)	422 (70.2)	Reference	

RA: rheumatoid arthritis; OR: odds ratio; CI: confidence interval; NA: not available. ^∗^Statistically significant (*P* < 0.05).

**Table 3:** Association of *SIGIRR rs7396562* polymorphism with laboratory parameters in patients with RA.

Variable	Genotype frequency, *n* (%)			*χ* ^2^	*P* value	Allele frequency, *n* (%)		*χ* ^2^	*P* value
rs7396562	TT	TG	GG			T	G		
RF									
Positive	155 (38.9)	178 (44.7)	65 (16.4)	1.505	0.471	488 (61.3)	308 (38.7)	1.401	0.237
Negative	38 (34.9)	48 (44.0)	23 (21.1)			124 (56.9)	94 (43.1)		
Anti-CCP									
Positive	157 (37.6)	184 (44.2)	76 (18.2)	1.478	0.478	498 (59.7)	336 (40.3)	1.602	0.206
Negative	28 (31.8)	40 (45.5)	20 (22.7)			96 (54.5)	80 (45.5)		

RA: rheumatoid arthritis; RF: rheumatoid factor; Anti-CCP: anticyclic citrullinated peptide. ^∗^Statistically significant (*P* < 0.05).

**Table 4:** Significant cell-specific cis-eQTL of *rs7396562* in eQTL base.

SNP	Chr	Position	eQTL gene	Tissue	*P* value
rs7396562	11	408353	SIGIRR	Blood	6.420*E* − 188

## Data Availability

Data can be obtained by contacting the corresponding author.

## References

[1] Kurkó J., Besenyei T., Laki J., Glant T. T., Mikecz K., Szekanecz Z. (2013). Genetics of rheumatoid arthritis—a comprehensive review. *Clinical Reviews in Allergy and Immunology*.

[2] Wu Q., Cao F., Tao J., Li X., Zheng S. G., Pan H.-F. (2020). Pentraxin 3: a promising therapeutic target for autoimmune diseases. *Autoimmunity Reviews*.

[3] Tobon G. J., Youinou P., Saraux A. (2010). The environment, geo-epidemiology, and autoimmune disease: rheumatoid arthritis. *Journal of Autoimmunity*.

[4] Viatte S., Plant D., Raychaudhuri S. (2013). Genetics and epigenetics of rheumatoid arthritis. *Nature Reviews Rheumatology*.

[5] Wang C., Feng C.-C., Pan H.-F., Wang D.-G., Ye D.-Q. (2013). Therapeutic potential of SIGIRR in systemic lupus erythematosus. *Rheumatology International*.

[6] Molgora M., Barajon I., Mantovani A., Garlanda C. (2016). Regulatory role of IL-1R8 in immunity and disease. *Frontiers in Immunology*.

[7] Polentarutti N., Rol G. P., Muzio M. (2003). Unique pattern of expression and inhibition of IL-1 signaling by the IL-1 receptor family member TIR8/SIGIRR. *European Cytokine Network*.

[8] Molgora M., Supino D., Mantovani A., Garlanda C. (2018). Tuning inflammation and immunity by the negative regulators IL-1R2 and IL-1R8. *Immunological Reviews*.

[9] Al-Kuhlani M., Lambert G., Pal S., de la Maza L., Ojcius D. M. (2020). Immune response against Chlamydia trachomatis via Toll-like receptors is negatively regulated by SIGIRR. *PLoS One*.

[10] Drexler S. K., Kong P., Inglis J. (2010). SIGIRR/TIR-8 is an inhibitor of Toll-like receptor signaling in primary human cells and regulates inflammation in models of rheumatoid arthritis. *Arthritis and Rheumatism*.

[11] Huang X., Hazlett L. D., Du W., Barrett R. P. (2006). SIGIRR promotes resistance against Pseudomonas aeruginosa keratitis by down-regulating type-1 immunity and IL-1R1 and TLR4 signaling. *Journal of Immunology*.

[12] Bulek K., Swaidani S., Qin J. (2009). The essential role of single Ig IL-1 receptor-related molecule/Toll IL-1R8 in regulation of Th2 immune response. *Journal of Immunology*.

[13] Gulen M. F., Kang Z., Bulek K. (2010). The receptor SIGIRR suppresses Th17 cell proliferation via inhibition of the interleukin-1 receptor pathway and mTOR kinase activation. *Immunity*.

[14] Thomassen E., Renshaw B. R., Sims J. E. (1999). Identification and characterization of SIGIRR, a molecule representing a novel subtype of the IL-1R superfamily. *Cytokine*.

[15] Zhu Y., Wang D.-G., Yang X.-K. (2014). Emerging role of SIGIRR rs7396562(T/G) polymorphism in systemic lupus erythematosus in a Chinese population. *Inflammation*.

[16] Petrackova A., Horak P., Radvansky M. (2019). Cross-disease innate gene signature: emerging diversity and abundance in RA comparing to SLE and SSc. *Journal of Immunology Research*.

[17] Petrackova A., Horak P., Radvansky M. (2020). Revealed heterogeneity in rheumatoid arthritis based on multivariate innate signature analysis. *Clinical and Experimental Rheumatology*.

[18] Wang L., Wang Y., Xia L., Shen H., Lu J. (2018). Elevated frequency of IL-37- and IL-18R*α*-positive T cells in the peripheral blood of rheumatoid arthritis patients. *Cytokine*.

[19] Arnett F., Edworthy C. (1988). The American Rheumatism Association 1987 revised criteria for the classification of rheumatoid arthritis. *Arthritis and Rheumatism*.

[20] Wang D.-Y., Su C., Chen G.-M. (2015). The decreased frequency of SIGIRR-positive CD4+ T cells in peripheral blood of patients with SLE and its correlation with disease activity. *Molecular Biology Reports*.

[21] Zhu Y.-Y., Su Y., Li Z.-G., Zhang Y. (2012). The largely normal response to Toll-like receptor 7 and 9 stimulation and the enhanced expression of SIGIRR by B cells in systemic lupus erythematosus. *PLoS One*.

[22] Batliwalla F. M., Li W., Ritchlin C. T. (2005). Microarray analyses of peripheral blood cells identifies unique gene expression signature in psoriatic arthritis. *Molecular Medicine*.

[23] Ishigaki K., Akiyama M., Kanai M. (2020). Large-scale genome-wide association study in a Japanese population identifies novel susceptibility loci across different diseases. *Nature Genetics*.

[24] Li P., Wang X., Zhao M.-Q. (2016). TCR-CD3*ζ* gene polymorphisms and expression profile in rheumatoid arthritis. *Autoimmunity*.

[25] Leng R. X., Di DS N. J., Wu X. X. (2020). Identification of new susceptibility loci associated with rheumatoid arthritis. *Annals of the Rheumatic Diseases*.

